# Bodyweight distribution between limbs, muscle strength, and proprioception in traumatic transtibial amputees: a cross-sectional study

**DOI:** 10.6061/clinics/2021/e2486

**Published:** 2021-04-16

**Authors:** Carlos Henrique da Silva Fontes, Conrado Torres Laett, Ubiratã Faleiro Gavilão, José Carlos de Campos, Dângelo José de Andrade Alexandre, Victor R.A. Cossich, Eduardo Branco de Sousa

**Affiliations:** I Centro de Amputados, Unidade de Reabilitacao, Instituto Nacional de Traumatologia e Ortopedia (INTO), Rio de Janeiro, RJ, BR.; II Laboratorio de Pesquisa Neuromuscular, Divisao de Pesquisa, Instituto Nacional de Traumatologia e Ortopedia (INTO), Rio de Janeiro, RJ, BR.; III Laboratorio de Biomecanica, Escola de Educacao Fisica e Desportos (EEFD), Universidade Federal do Rio de Janeiro (UFRJ), Rio de Janeiro, RJ, BR.; IV Programa de Pos-graduacao em Ciencia da Reabilitacao, Centro Universitario Augusto Motta (UNISUAM), Rio de Janeiro, RJ, BR.; V Area de Fisioterapia, Instituto Nacional de Traumatologia e Ortopedia (INTO), Rio de Janeiro, RJ, BR.; VI Divisao de Ensino e Pesquisa, Instituto Nacional de Traumatologia e Ortopedia (INTO), Rio de Janeiro, RJ, BR.

**Keywords:** Body Weight, Amputation, Muscle Strength, Proprioception, Position Sense

## Abstract

**OBJECTIVES:**

To evaluate how transtibial amputation (TT) affects bodyweight distribution, voluntary knee joint position sense (JPS), and quadriceps (QUA) and hamstrings (HAM) strength in prosthetized patients.

**METHODS:**

Only TT patients who had been prosthetized for more than one year were included, and an age-paired able-bodied group was used as control. The participants stood on force plates with their eyes open to measure bodyweight distribution between the limbs. Knee voluntary JPS was assessed by actively reproducing a set of given arbitrary joint angles using a video analysis approach, and QUA and HAM strength were assessed isometrically with a hand-held dynamometer.

**RESULTS:**

Sixteen TT subjects (age: 39.4±4.8 years) and sixteen age-paired control subjects (age: 38.4±4.3 years) participated in the study. The amputees supported their bodyweight majorly on the sound limb (54.8±8.3%, *p*<0.001). The proprioceptive performance was similar between the amputated (absolute error (AE): 2.2±1.6°, variable error (VE): 1.9±1.6°, constant error (CE): -0.7±2.0°) and non-amputated limbs (AE: 2.6±0.9°, VE: 2.1±0.9°, CE: 0.02±2.3°), and was not different from that of control subjects (AE: 2.0±0.9°, VE: 1.4±0.4°, CE: -1.1±1.7°). There was a considerable weakness of the QUA and HAM in the amputated limb compared with the sound limb and control subjects (*p*<0.001 both).

**CONCLUSIONS:**

The asymmetric bodyweight distribution in the transtibial amputees was not accompanied by a reduction in knee proprioception. There was significant weakness in the amputated limb, which could be a potential issue when designing rehabilitation programs.

## INTRODUCTION

Lower limb amputation is a life-changing event that modifies from basic locomotion (1,2) to social and psychological aspects (3). Approximately 40-80% of lower limb amputations are due to vasculopathy and diabetes (4,5). However, in younger individuals, lower limb loss occurs mainly due to traumatic events, such as road traffic or gunshot injuries (5,6). Trauma-related amputations are the primary etiology in developing countries and represent a substantial cause of amputation worldwide (4,5). Despite the limitations imposed on the patient, transtibial amputation (TT), at least when performed at the distal third of the lower leg, enables the patient to have a physically active lifestyle after proper prosthetic fitting and rehabilitation (7,8).

Limb amputation was previously associated with thigh muscle atrophy and fat permeation in the stump compared with the sound limb and able-bodied subjects (9), modified knee joint kinematics during gait and stair climbing (1,10,11), and an augmented muscle voluntary activation of the biceps femoris—measured as the integrated surface electromyography signal—during gait (8).

Several studies have investigated the muscle strength of TT patients (12-17). Besides the methodological differences—isokinetic *vs.* isometric evaluation, different velocities, and devices—in general, the studies suggested a reduced strength of both knee extensors and flexors in comparison with the sound limb (12-17). This is a clinically relevant finding, given that thigh muscle weakness could lead to poor performance during standing and gait, thus compromising the execution of daily functional tasks (12).

The TT procedure leads to loss of all muscles, tendons, ligaments, and articular capsule, seriously affecting the lower limb, and consequently, body function (9,18). These structures are essential not only for locomotion but they also function as sensory receptors, which, when absent, may compromise proprioception (9,18). Conscious proprioception involves kinesthesia, force sense, and joint position sense (JPS) (19). Few studies have investigated proprioception alterations in TT patients and they have presented different results (20-22). These conflicting findings are probably due to different methodological approaches (*i.e.*, joint angular reproduction and passive movement detection) and not to standardized amputation procedures (23). Although the knee joint capsule is kept intact, the interaction between the stump and the prosthetic limb may alter the JPS by merely changing the effort needed to move the limb due to changes in its mass (24). JPS can be assessed using both involuntary (passive) and voluntary movements, the latter being more useful because it mimics the joint motion in real-life situations (25,26).

Bodyweight is supported by the non-amputated (NAMP) limb in the orthostatic position, not only at rest (27-29) but also during gait (30), increasing the mechanical load at its joints (12-17). At early rehabilitation stages, the NAMP limb supports up to 60% of the bodyweight (27-29,31). This asymmetrical weight distribution also produces balance changes, which increase the risk of falling, while the higher mechanical demand placed on the sound limb is suggested as the main factor for osteoarthritis development and the overuse symptoms observed in TT patients (32,33).

A better understanding of neuromuscular changes due to traumatic TT is paramount to the development of comprehensive and meaningful clinical interventions. Although changes in bodyweight distribution after TT are well documented (27-29), few studies have aimed to assess voluntary knee JPS (21) and knee extensor and flexor strength together, therefore, justifying further investigations. Thus, the goals of this study were to verify the effect of TT on bodyweight distribution, voluntary knee JPS, and quadriceps (QUA) and hamstrings (HAM) strength in prosthetized patients. As some intervening variables (*i.e.*, stump length, age, and time from prosthesis use) could influence the outcome, we chose to control those variables. We hypothesized that alterations in bodyweight distribution, knee JPS, and QUA and HAM strength should be observed when compared with the sound limb and with age-paired healthy subjects.

## MATERIALS AND METHODS

### Participants

The TT group included patients aged between 18 and 50 years, who underwent unilateral amputation due to traumatic etiology, and had been using the prosthesis for at least one year. Patients were excluded if they were diabetic, reported previous knee injury or surgery in any of the limbs, or presented with neurological, vascular, vestibular, or visually diagnosed disorders. All patients wore the same standard lower limb endoskeletal prosthesis (Ethnos, Brazil) with a dynamic foot (Ossur, Iceland). Control subjects were age-paired and recruited from the staff of the hospital’s rehabilitation division. The Tegner score was used to measure work level, physical activity, and sport-related activity engagement in both groups (34), and the dominant (DON) leg was self-defined as the preferred kicking leg (Table 1). This study was approved by the hospital’s ethics committee (process #01128818.0.0000.5273). All volunteers were previously informed about the procedures and goals of the study and they provided written consent to participate.

### Procedures

This was an analytical, controlled cross-sectional with control group study. All the procedures were performed during a single testing session, lasting no longer than two hours, on a day that the patient usually had a routine medical screening scheduled (conducted between April-June 2019). First, the participants were asked to complete the bodyweight distribution test, followed by knee extension JPS, and by maximal voluntary isometric contractions of QUA and HAM. Bodyweight distribution was measured while the subject wore the prosthetic leg. On other hand, JPS and muscle strength evaluations were conducted on both limbs (without the prosthesis). All devices and methods used were highly reliable, as reported elsewhere (35-37).

### Assessment of bodyweight distribution

We used a set of two separate 24×40 cm force plates (Globus Ergo System, Italy) to quantify bodyweight distribution. Volunteers were asked to stand still in an orthostatic position with one foot on each force plate for 60s. They were then instructed to look forward to a fixed point at the eye level on the wall, 2m away. This was performed three times, with a 30s rest period between each measurement. Ground reaction force was calculated as an average of the three measurements for each limb, and bodyweight distribution was defined as a percentage of the total ground reaction force.

### Assessment of proprioception

**Figure 1:**
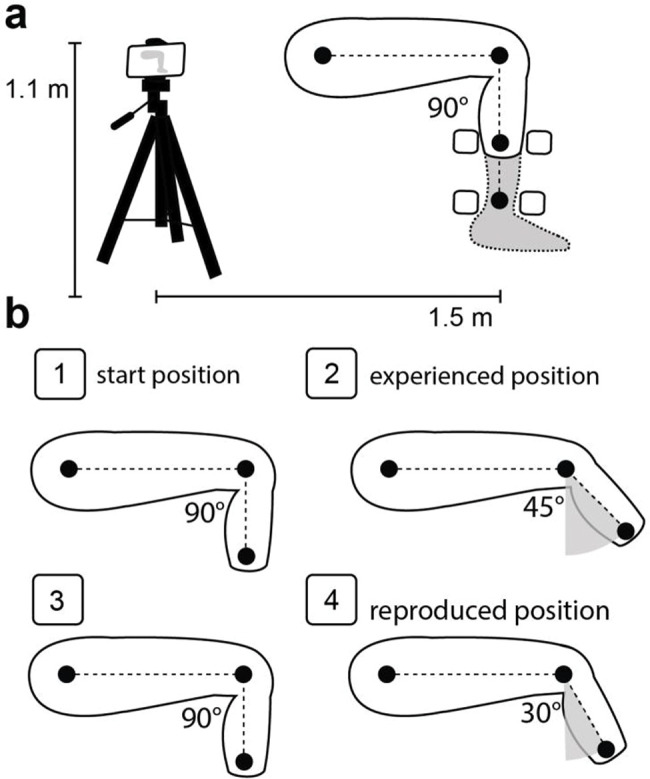
Overview of the experimental conditions. a) Representative set-up to video recording during the joint position sense and strength evaluation. The black circles represent the Styrofoam balls used to measure distance and angles. The squares represent the positions used to set the hand-held dynamometer. b) Step-by-step representation of the joint position sense, b-1: demonstrated the start position ∼90° of knee flexion, b-2: the experienced position, b-3: the subject returning to the start position and waiting for the evaluator’s subsequent commands, b-4: the reproduced position. The difference between the reproduced and experienced positions were used for calculating proprioception indices. The angles 45° and 30° are example values.

Knee proprioception was assessed using JPS. The subjects were seated on a chair with their backs entirely supported, their hips flexed at approximately 90° (0°=full extension), and their lower legs hanging freely (Figure 1). The test consisted of the experience and reproduction of a joint position through voluntary movements with the participants blindfolded, as described in a previous study (26). Briefly, the test began with the volunteers’ lower legs hanging relaxed, then the evaluator asked the volunteers to slowly extend their knees until the command to “stop” and keep that position for three seconds (experienced position). Then, the evaluator asked the volunteers to “return to the start position and relax,” and after 3 seconds of rest, the command “show me where your knee was before” was given, so the volunteers slowly extended their knees, trying to reach the experienced position (reproduced position) while holding their limbs for more than 3s. This procedure was performed four times for each limb after three unrecorded familiarization trials, and the knee angles tested were randomly assigned. All procedures were video-recorded on the sagittal plane using a smartphone camera (Motorola Moto X Play, 21 Mpx, Full HD). The camera was positioned 1.1m from the ground level in a stable tripod kept 1.5m from the subject. Styrofoam balls were fixed at the hip, femur lateral epicondyle, and at the most distal portion of the stump or in the malleolus of the fibula (Figure 1). The knee joint angle was measured from the footage using ImageJ software (National Institutes of Health, Bethesda, Maryland, USA). The difference between the reproduced and experienced positions was used to calculate the absolute error (AE), variable error (VE), and constant error (CE), where AE corresponds to the absolute average, VE to the standard deviation, and CE to the average of the attempts (25,26).

### Assessment of muscle strength

We used a hand-held dynamometer (Nicholas MMt Handheld Dynamometer, Model 001160, Lafayette Instrument Company, USA) to assess the QUA and HAM muscle strength. The participants were positioned in the same way as for the JPS test. The dynamometer was positioned anteriorly or posteriorly on the most distal portion of the stump/leg (Figure 1a). For each test, volunteers were asked to isometrically perform the maximum voluntary contraction (MVC) against the dynamometer, which was held with the aid of an inelastic strap. MVC was conducted with the knee flexed at 90° (0°=knee fully extended) and kept for at least 5s. Three attempts were performed with a 30s rest period between each, and only the maximum value was used for the analyses. The test order between muscle groups and limbs was randomized, and before each test, three submaximal trials (50%, 75%, and 100% of perceived maximum effort) were performed for familiarization and warm-up. All procedures were video-recorded, and images were used to measure the lever arm to calculate the joint torque (product of force by the lever arm). The lever arm was defined as the distance between the femoral lateral epicondyle and the most distal part of the stump or limb where the dynamometer was positioned. The peak torque value was recorded for further statistical analyses.

### Statistical analysis

Data were presented as mean±SD. Data distribution was verified and classified as normal (*p*>0.05) using the Shapiro-Wilk test. First, the DON and non-dominant (NDON) limbs of the control group were compared using Students *t*-test for independent samples to evaluate if the DON limb could be used for subsequent analysis as the control limb (CTL). One-way ANOVA for independent samples was used to compare the amputated (AMP) and NAMP limbs with the CTL. The Bonferroni *post-hoc* test was adopted when needed to verify the differences between pairs of limbs. The effect size was assessed by *η* ^2^ *_p_* and classified as small (0.01), medium (0.06), and large (0.14). Statistical procedures were performed using the SPSS software (IBM SPSS Inc., version 19, Chicago, IL, USA), and data were considered statistically significant at *p*≤0.05.

## RESULTS

**Figure 2:**
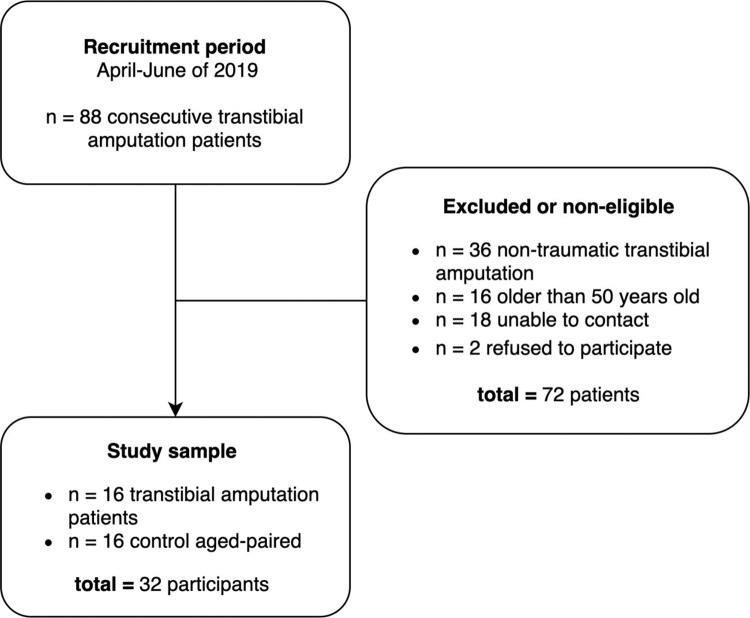
A flow diagram of the recruitment, total eligible participants, and the final sample in the study.

A total of 88 consecutive patients were selected from the hospital’s amputee center rehabilitation program. The first attempt of contact with the patients was made by a standardized telephone call (conducted by CHSFF) between April and June 2019 to collect preliminary data and ask for interest in participating in the study. Of the total, 36 patients had a TT with non-traumatic etiology, 16 were older than the pre-defined inclusion age range, 18 of the patients could not be reached, and two subjects refused to participate in the study. A total of 16 TT patients and 16 control subjects participated in this study (Figure 2).

We did not observe differences in the demographic characteristics between the amputees and control groups (Table 1). None of the TT required walking aids (*e.g.*, cane or crutches), reported phantom limb, or stump pain. Also, we did not observe significant differences between the DOM and NDOM limbs of the control group for any studied variable: bodyweight distribution (mean±SD, DOM: 37.4±6.8 kg, NDOM: 37.7±7.1 kg, *p*=0.88), AE ( DOM: 2.0±0.9°, NDOM: 2.6±1.1°, *p*=0.98), VE (DOM: 1.4±0.4°, NDOM: 1.8±0.8°, *p*=0.92), CE (DOM: -1.1±1.7°, NDOM: -1.9±1.8°, *p*=0.22), QUA peak torque (DOM: 112.3±26.5 Nm, NDOM: 120.0±29.4 Nm, *p*=0.44), and HAM peak torque (DOM: 55.1±16.3 Nm, NDOM: 55.3±9.5 Nm, *p*=0.98). The TT group demonstrated an asymmetrical bodyweight distribution, with more weight on the NAMP limb (Table 2). We did not observe significant differences in any of the proprioception measures between the limbs (Table 2). For both QUA and HAM peak torque, the AMP limb demonstrated a lower value than the NAMP and CTL (Table 2).

## DISCUSSION

The goals of this study were to verify the effect of TT on bodyweight distribution, proprioception, and thigh muscle strength. The main findings were that amputee patients demonstrated an asymmetrical bodyweight distribution, with no differences in knee JPS. Nevertheless, both the QUA and HAM muscles were weaker in the amputated limb than in the sound limb and control subjects.

Lower limb amputation imposes changes on the body’s mechanical behavior during tasks in standing position, characterized by altered kinematics of ambulation (1,10,11) and obstacle crossing (2). An asymmetrical weight distribution increases the mechanical load on the joint surface, which is a factor for osteoarthritis (17,38). Our results confirm the previously reported asymmetrical bodyweight distribution (27-29), with the prosthetic limb usually carrying approximately 45% of the total bodyweight (28,29). Previously, pain, discomfort, and insecurity have been suggested as factors for bodyweight asymmetry (39). However, since none of our TT patients reported pain, discomfort on the stump-prosthetic interface, or insecurity, we cannot directly link those factors with the bodyweight distribution results. Although we cannot discard that at a subconscious level, patients still have unreported issues.

We did not observe differences in JPS between the AMP and NAMP limbs, as with the control subjects. This corroborates previous studies that also did not observe proprioception deficits in amputee patients (20,21). It is expected that TT results in the loss of entire populations of mechanoreceptors during both the traumatic event and the surgery. Theoretically, a decrease in the number of mechanoreceptors alone is sufficient for proprioception impairment. Moreover, joint capsule mechanoreceptors are considered the main structures responsible for measuring joint motion, and voluntary muscle contraction allows the assessment of sensory information coming from the muscle spindles and Golgi tendon organs (24). We suggest that the lack of proprioception deficits can be explained by maintaining the knee joint structures (*i.e.*, joint capsule, ligaments, and tendons) and by the voluntary muscle actions used during the JPS test.

Previous observations have reported weaker QUA and HAM on the AMP limb (12,13,17). Loyd et al. (17) analyzed subjects with multi-cause amputations, ranging from trauma to peripheral vascular diseases. However, given the high coefficient of variation (approximately 60%), the effects of comorbidities and advanced age of the non-traumatic patients confounded the results. Isakov et al. (12) used an adapted isokinetic dynamometer device to measure knee isometric extension and flexion of TT amputees of unreported etiology, reporting values similar to ours (QUA: 35.6±25.4, and HAM: 25.1±13.5 Nm). Moirenfeld et al. (13) also used a standard isokinetic device to measure the QUA and HAM strength (velocity of 120°/s) of traumatic TT amputees. They reported that the patients used the prosthesis during the test to allow proper fitting of the device, thus reducing the QUA and HAM strengths to 50% and 35%, respectively, compared with the sound limb.

The hand-held dynamometer used in this study has already been demonstrated to be a feasible and reliable method to measure muscle strength (36). This approach was adopted because the stump length did not fit the standard isokinetic dynamometer device. In addition, we preferred to measure strength without the prothesis to directly assess the subject’s limb, preventing torque production between the prosthesis and the stump, which could be painful and generate biased results. Interestingly, the QUA seems to be more affected than the HAM, corroborating previous observations (13). The QUA and HAM were, respectively, 3.8 and 2.6 times stronger in the sound limb than in the AMP limb. We hypothesized that this smaller difference in HAM muscle strength could emerge from the prosthesis weight working as resistance to knee flexion during ambulation (1,10,11). This resistance probably produces an increase in the mechanical demand of the HAM, reducing the strength impairment. Although these ideas are solely speculative, the atrophy of the QUA was previously reported to be greater than that in the semitendinosus muscle (16-31% *vs*. 5%) (40), reinforcing this idea. However, future investigations should be performed to confirm this.

This is the first study that concomitantly assessed bodyweight distribution, knee active JPS, and QUA and HAM strength in traumatic TT amputees. Together, the tests provide more information about the clinical status of these patients, which may help physicians and physical therapists in the development of new rehabilitation programs. This study presents as strong points that all patients presented with the same etiology (traumatic), which influenced the surgical technique, and wore the same kind of prosthetic limb, which influenced gait pattern. In addition, we used standardized, replicable, and widely reported methods for all tests (25,26,35-37), allowing experimental set replication even in a clinical environment. Moreover, the present study had a larger sample size than other studies (20-22). On the other hand, the fact that patients underwent amputation and immediate postoperative rehabilitation in different centers are weak points, although these were minimized by the fact that all patients received the same pre- and immediate post-prosthetic rehabilitation protocols. Although the patients did not demonstrate proprioceptive deficits, this result should be considered with caution. We only evaluated a single conscious proprioception sub-modality, and we are unaware of studies that evaluated the sense of force in this population. The improvement of lower limb strength should be continuously addressed in this population, which probably will affect in the bodyweight asymmetry and gait patterns. We evaluated only post-traumatic amputees, well-adapted to wearing a prosthesis for a long time, without pain, discomfort, or any rehabilitation-related problems. Thus, the results should be analyzed in terms of the sample characteristics and not by generalization.

In conclusion, bodyweight was asymmetrically distributed between the AMP and NAMP limbs. However, no alterations in the ability to sense and reproduce knee joint angles were demonstrated using an active test. QUA and HAM weakness observed in the AMP limb suggests that, although the patients were wearing the prosthetic limb for a long time, their strength level was still low.

## AUTHOR CONTRIBUTIONS

Fontes Filho CHS was responsible for the study conception, data collection, manuscript writing, review and approval of the final version. Laett CT was responsible for the data collection and analysis, manuscript writing, review and approval of the final version. Gavilão UF was responsible for the data collection and analysis, manuscript review and approval of the final version. Campos Jr JC was responsible for the data collection and analysis, manuscript review and approval of the final version. Alexandre DJA was responsible for the study conception, manuscript review and approval of the final version. Cossich VRA was responsible for the study conception, data collection and analysis, manuscript writing, review and approval of the final version. Sousa EB was responsible for the study conception, manuscript writing, review and approval of the final version.

## Figures and Tables

**Table 1:** Demographic characteristics of the amputees and control groups.

Variable	Amputees (n=16)	Control (n=16)	*p*-value
**Age (years)**			
mean (SD)	39.4 (4.8)	38.4 (4.3)	0.55
Minimum	31	31	
Maximum	48	47	
**Sex, number (%)**			
Men	11 (6.0)	9 (56.0)	NA
Women	5 (31.0)	7 (44.0)	
**Weight (kg)**			
mean (SD)	81.3 (22.2)	72.1 (14.0)	0.17
Minimum	48	52	
Maximum	125	98	
**Height (m)**			
mean (SD)	1.70 (0.10)	1.70 (0.10)	0.24
Minimum	1.53	1.56	
Maximum	1.82	1.81	
**BMI (kg/m^2^)**			
mean (SD)	27.3 (6.0)	25.5 (3.6)	0.31
Minimum	20.4	19.6	
Maximum	38.6	33.5	
**Amputated limb, number (%)**			
Right	10 (62.5)	NA	NA
Left	6 (37.5)	NA	
**Dominance, number (%)**			
Right	14 (87.5)	16 (100)	
Left	2 (12.5)	0 (0)	
**Tegner score (pts)**			
mean (SD)	3.6 (1.9)	4.8 (2.0)	0.12
Minimum	1	3	
Maximum	7	9	

BMI: body mass index. NA: not applicable. The *p*-value were obtained using t-tests for independent measures.

**Table 2:** Bodyweight distribution, proprioception, and muscular strength in amputees and control subjects.

Variable	AMP	NAMP	CTL	*p*-value	*η* ^2^ *_p_*
Bodyweight distribution (%)	45.2±8.3*	54.8±8.3	49.7±2.3	<0.001	0.25
AE (°)	2.2±1.6	2.6±0.9	2.0±0.9	0.39	0.04
VE (°)	1.9±1.6	2.1±0.9	1.4±0.4	0.13	0.08
CE (°)	-0.7±2.0	0.02±2.3	-1.1±1.7	0.26	0.06
QUA peak torque (Nm)	31.6±13.3^#^	119.4±56.2	112.3±26.5	<0.001	0.56
HAM peak torque (Nm)	23.5±10.7^#^	61.5±26.1	55.1±16.3	<0.001	0.46

AMP, amputated limb. NAMP, non-amputated limb. CTL, control limb (control group dominant leg). AE, absolute error. VE, variable error. CE, constant error. QUA, quadriceps. HAM, hamstrings. *Significantly different from NAMP. ^#^Significantly different from the NAMP and CTL groups. The *p*-values were obtained using one-way ANOVA. The *η*^2^*_p_* represents the effect size measure.
